# Genomic methods reveal independent demographic histories despite strong morphological conservatism in fish species

**DOI:** 10.1038/s41437-021-00455-4

**Published:** 2021-07-05

**Authors:** Jessika M. M. Neves, Zachary J. Nolen, Nidia N. Fabré, Tamí Mott, Ricardo J. Pereira

**Affiliations:** 1 Instituto de Ciências Biológicas e da Saúde, Universidade Federal de Alagoas, Maceió, Alagoas Brazil; 2 Division of Evolutionary Biology, Faculty of Biology II, Ludwig-Maximilians-Universität München, Grosshaderner Strasse 2, Planegg-Martinsried, Germany; 3 Department of Biology, Lund University, Lund, Sweden

**Keywords:** Genetic variation, Phylogenetics, Speciation

## Abstract

Human overexploitation of natural resources has placed conservation and management as one of the most pressing challenges in modern societies, especially in regards to highly vulnerable marine ecosystems. In this context, cryptic species are particularly challenging to conserve because they are hard to distinguish based on morphology alone, and thus it is often unclear how many species coexist in sympatry, what are their phylogenetic relationships and their demographic history. We answer these questions using morphologically similar species of the genus *Mugil* that are sympatric in the largest coastal Marine Protected Area in the Tropical Southwestern Atlantic marine province. Using a sub-representation of the genome, we show that individuals are assigned to five highly differentiated genetic clusters that are coincident with five mitochondrial lineages, but discordant with morphological information, supporting the existence of five species with conserved morphology in this region. A lack of admixed individuals is consistent with strong genetic isolation between sympatric species, but the most likely species tree suggests that in one case speciation has occurred in the presence of interspecific gene flow. Patterns of genetic diversity within species suggest that effective population sizes differ up to two-fold, probably reflecting differences in the magnitude of population expansions since species formation. Together, our results show that strong morphologic conservatism in marine environments can lead to species that are difficult to distinguish morphologically but that are characterized by an independent evolutionary history, and thus that deserve species-specific management strategies.

## Introduction

Human overexploitation of natural resources has placed conservation and management as one of the most pressing challenges in modern societies (Mora and Zapata 2013). Over the past four decades, heavy targeting by fisheries has contributed to a decline in average population size of more than 36% and 81% in marine and freshwater ecosystems, respectively (WWF 2016). Understanding the evolutionary history of impacted species is fundamental for establishing strategies to protect and sustain biological diversity (Crandall et al. 2000; Moritz 2002; Cook and Sgrò 2019). Nevertheless, this task is particularly challenging in morphologically conserved species because they are difficult to distinguish based on external morphology alone, requiring an integrated approach between morphological and molecular tools that until recently have been unavailable for non-model organisms (da Fonseca et al. 2016).

Understanding the number and distribution of morphologically cryptic species has strongly benefited from studying fast evolving mitochondrial genes, which are revealing an exponentially growing number of species (Sáez and Lozano 2005), and their demographic history (Carnaval et al. 2009). Such discoveries of hidden genetic diversity have been reported even in previously known biodiversity hotspots, such as in the Amazon (Benzaquem et al. 2015), and in marine ecosystems (Asgharian et al. 2011; Brandão et al. 2016). Although many cryptic species have allopatric distributions (Wake 2009), many others are partially or fully sympatric (McBride et al. 2009; Moritz et al. 2018), suggesting that cryptic speciation can result in stable genetic boundaries between species. Thus, assessing the phylogenetic relationships between sympatric cryptic species, their levels of genetic connectivity, and their demographic history offer important insights into the process of species formation.

Although mitochondrial studies have been fundamental in identifying morphologically conserved species and mapping their distribution (Ward et al. 2009), mitochondrial markers are limiting when assessing demographic history (Galtier et al. 2009). To better inform sustainable conservation measures, it is thus necessary to use hundreds or thousands of independent nuclear markers to accurately reconstruct the demographic history of these species (Grewe et al. 2015; Allendorf 2017; Grundler et al. 2019). Recent advances in sequencing technology and statistical methods now offer unprecedented opportunities for the field of conservation biology across taxa, providing new insights into the adaptive capacity in tortoises (Scott et al. 2020), genetic connectivity in terrestrial mammals (Pedersen et al. 2018), and historical changes in effective population size in marine mammals (Peart et al. 2020; Bilgmann et al. 2021).

To infer the evolutionary processes underlying the genetic diversity of sympatric cryptic marine fishes, we focus on the genus *Mugil*, commonly known as mullets. Historically, these species have been heavily targeted by traditional and industrial fisheries (Whitfield et al. 2012; Pacheco-Almanzar et al. 2017)—with total harvest reaching about 140 k tons globally in 2013 (Crosetti 2016) and significant decreases in census sizes within the last 25 years (Mendonça and Bonfante 2011; Sant’Ana et al. 2017; Vieira et al. 2019). Nevertheless, their diversity and evolutionary history is just starting to be revealed by molecular studies (Durand and Borsa 2015; Xia et al. 2016; Delrieu-Trottin et al. 2020; Neves et al. 2020).

**Fig. 1:**
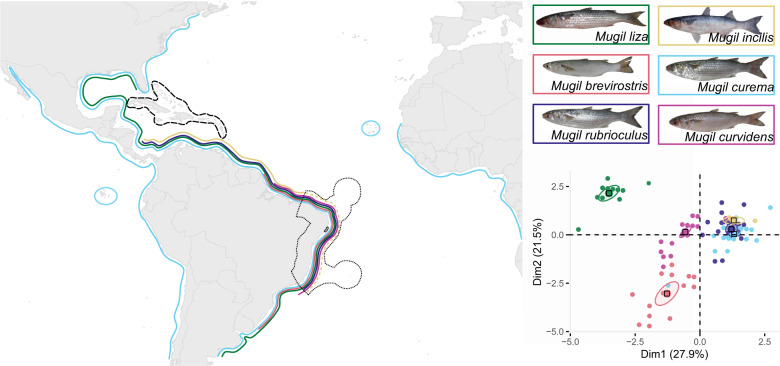
Distribution of the six species of *Mugil* fishes that are potentially sympatric at the Coral Coast MPA (highlighted in doted black line). Each depicted species is surrounded by a rectangle with color matching the species distribution (Menezes et al. 2015; Barletta and Dantas 2016; Durand and Whitfield 2016; Pacheco-Almanzar et al. 2016). Dashed yellow line indicates the area where the occurrence of *M. incilis* has been debated (Menezes et al. 2015). Dashed black line indicates that individuals of all species except *M. brevirostris* may occur. *Mugil incilis* photo by A. Carvalho. The plot on the right corner represents a multiple factor analysis based on morphological traits (Neves et al. 2021); ellipses represent 95% confidence level.

*Mugil* species live in fresh and brackish waters during most of their life cycle, migrating to the sea to reproduce (Nordlie 2016). Thus, they play a fundamental role in transferring energy between estuaries and coastal systems (Lebreton et al. 2011), helping in the maintenance of biological productivity and, consequently, the yield of other fisheries. Several morphologically similar species of *Mugil* occur sympatrically in tropical and subtropical waters, making it necessary to use genetic information to better understand the number of species and their distribution (Durand et al. 2012b; Durand and Borsa 2015; Xia et al. 2016; Neves et al. 2021). Such challenges in assessing species richness and diversity also occur within important Marine Protected Areas (MPAs) designed specifically to protect species of high ecologic and economic importance, such as the Coral Coast MPA, the largest coastal Tropical Southwestern marine province (de Souza et al. 2012). In this MPA, sympatric species of *Mugil* are extremely challenging to identify based on current taxonomic keys (Menezes et al. 2015), resulting in up to 14% of individuals receiving conflicting morphological and mitochondrial classifications (Neves et al. 2021). Most of the conflicts occur in individuals that carry the mitochondrial haplotype associated with *M. curema* but are morphologically assigned to *M. rubrioculus*, *M. incilis*, or to *M. curvi*dens (Neves et al. 2021). Although *M. rubrioculus* and *M. curema* are remarkably similar in external morphology (Fig. 1), they have diverged ~29 million years ago (mya) (Neves et al. 2020), reflecting a strong morphological conservatism in the evolution of these species. Such morphological similarity leads to strong disagreements among biologists regarding the number and distribution of these species (Durand et al. 2012a; Menezes et al. 2015; Pacheco-Almanzar et al. 2016) and reinforces the difficulties in establishing species-specific regulations for fisheries. Currently, *Mugil* species are targeted by traditional fisheries throughout the MPA without species-specific quotas, although we lack information regarding their relative abundance.

Here, we use double-digest restriction of genomic DNA associated with high-throughput sequencing to genotype thousands of markers across nominal species of *Mugil* that are sympatric in the Coral Coast MPA to: (i) determine the number of *Mugil* species in the area, (ii) quantify patterns of genetic connectivity within species across two estuaries and between sympatric species in the same estuary, and (iii) estimate the demographic history of every species. Our results provide new insights into the evolution of these species and provide guidelines for sustainable management.

## Materials and methods

### Sampling

#### Specimen collection

We used 94 muscle tissue samples of *Mugil* species that occur sympatrically in the Coral Coast MPA (Fig. 1) and that were classified as 6 morphological species (*Mugil liza, M. brevirostris*, *M. rubrioculus*, *M. curema, M. incilis*, and *Mugil curvidens*), according to the current taxonomic key (Menezes et al. 2015). A previous study (Neves et al. 2021) established that these individuals contained only five mitochondrial lineages, based on a diagnostic COI barcoding gene (Table S1), suggesting either an overestimation of species number or mitochondrial introgression. Here, we test the morphological and mitochondrial hypotheses using nuclear data.

While most specimens were collected in a partial reserve of the MPA where traditional fisheries exploit *Mugil* without restrictions (Santo Antonio estuary), 15 of the 30 individuals of *Mugil curvidens* were collected in an estuary 38 km away, where fisheries have some restrictions to protect manatee populations (Manguaba estuary; Fig. S1). We included this population to test whether populations of *M. curvidens* from different estuaries function as a single panmictic population, or whether measurable barriers to gene flow exist between these locations.

#### Genomic sequencing

Tissue samples were sent in a sequencing plate to DArT™ (Diversity Array Technology), who performed DNA extraction, tested two combinations of enzymes (*Pst*I/*Hpa*II and *Pst*I/*Sph*I), performed high-throughput sequencing for the best enzyme combination (*Pst*I/*Sph*I), assembled the loci, and called genotypes for the 94 individuals. This process was replicated for 32 samples (126 libraries generated in total), to estimate reproducibility and error rate of the genotypes (Grewe et al. 2015).

This method is analogous to the ddRAD protocol (Peterson et al. 2012), in the sense that two restriction enzymes (a rare and a common cutter) digest the genomes of individuals from closely related species in presumably homologous sites. Complementary Illumina adapters including individual barcodes are ligated to each restriction site, the libraries are size-selected, then amplified in 30 rounds of PCR. The resulting sequences are then processed using proprietary DArT analytical pipelines, which remove poor quality sequencing reads and demultiplex ~1,500,000 reads per individual. A secondary genotype-calling pipeline is applied to identify homologous clusters across individuals and retain clusters or loci with: a balanced counts per allele, a Mendelian distribution of alleles, and greater than 25X coverage.

Each individual was characterized by an array of SNPs, where *0* is homozygous for the major allele, *1* is heterozygous, *2* is homozygous for the minor allele, and *-* represents missing data. Each SNPs is characterized by: reproducibility (fraction of allele calls which are consistent among the technical replicates generated from the same DNA samples), call rate (the proportion of individuals scored for that locus), and polymorphism information content (PIC: index for evaluating the informative extent of a SNP marker, varying between zero for no allelic variation and 1.0 for maximum allele variation).

Because the focal species have diverged between ~29 to ~6 mya (Neves et al. 2020) and some have diverged in chromosome number and structure (Galetti et al. 2000; Nirchio et al. 2005, 2017; Rossi et al. 2005), it is likely that the restriction enzymes would not cut the same genomic regions across species. We tested for biases on the distribution of missing data by plotting the missing data per individual and the call rate per species, using the package dartR (Gruber et al. 2018) in R software (R Core Team 2020).

#### Data filtering

We used the package dartR (Gruber et al. 2018) to filter the data and to produce the input files for all downstream analyses (Table S3). We retained the SNPs with the following criteria: (1) SNPs with reproducibility above 97%, to reduce genotyping error; (2) only polymorphic SNPs; (3) loci (trimmed sequence tags) that are distinct enough to avoid paralogous loci (threshold: 0.2 of Hamming distance); (4) a varying amount of missing data (0, 20, and 40%, corresponding to call rates of 100, 80, and 60, respectively); and (5) one SNP per locus favoring SNPs with higher informativity (PIC values), to assure statistical independence among SNPs required by most analyses.

**Fig. 2:**
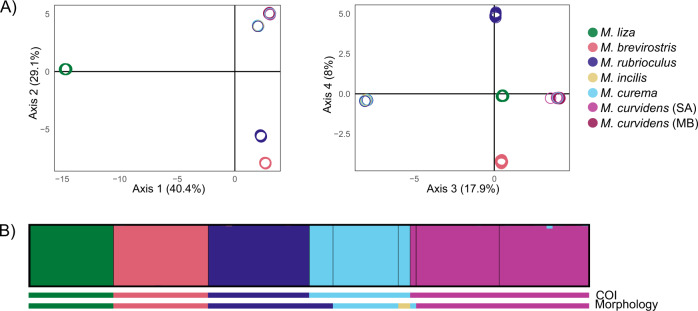
Population structure analyses performed with 94 *Mugil* individuals and the dataset with 0% missing data (984 SNPs). **A** PCoA analysis; **B** STRUCTURE analysis. In both analyses, individuals with disagreement between morphological and mitochondrial identification belong to the same mitochondrial lineages as recovered by the COI gene (Neves et al. 2021). There is no evidence of genetic population structure between the samples of *M. curvidens* from two estuaries (SA—Santo Antonio; MB—Manguaba).

Because missing data was not equally distributed among species (Fig. S2), for performing comparative analyses across the six *Mugil* species (hereafter “6sp”), we built datasets with the three levels of missing data (hereafter “0MD”, “20MD”, and “40MD). Because *M. liza* contained most of the missing data (Fig. S2), we repeated this process excluding *M. liza* without missing data (“5sp_0MD”). To estimate demographic history per species, we have produced 5 species-specific datasets with 40% of missing data, following the classifications based on mitochondrial and nuclear data, since these were strictly concordant (see Results; Fig. 2). We also produced a dataset with no missing data considering only *M. curvidens*, to test for population structure. See Table S4 for details on the various datasets and their use in corresponding analyses.

### Analyses

#### Population structure

In order to assess how many evolutionary lineages are present in our sampling, we performed two population structure analyses that differ in their model assumptions. First, we performed a non-model based Principal Coordinates Analysis (PCoA) to quantify how the genetic variance is distributed among samples based on the presence or absence of alleles, using the package dartR (Gruber et al. 2018) in R software (R Core Team 2020) and the three datasets for the six morphological species (6sp_0MD, 6sp_20MD, 6sp_40MD). Second, we estimated the number of genetic clusters and tested whether there is ongoing hybridization between them, using the algorithm implemented in STRUCTURE v.2.3.4 (Pritchard et al. 2000), which maximizes Hardy–Weinberg and Linkage equilibria within K ancestral clusters. We considered one to ten K clusters, with five replicates for each, 10 k iterations as burn-in, 10 k MCMC steps, independent allelic frequencies, and no prior on the assignment of individuals. We chose the most likely K based on log-likelihood values (Pritchard et al. 2000). The graphic output was built using Clumpak (Kopelman et al. 2015). We performed this analysis, using the most restrictive datasets containing all six species (6sp_0MD), the five species excluding *M. liza* (5sp_0MD), and *M. curvidens* alone (curvidens_0MD), to test whether there is population structure when maximizing the number of SNPs.

For both analyses, we expect to find evidence for six clusters if the morphological hypothesis is correct or for five clusters if the mitochondrial hypothesis is correct (Table S1; Neves et al. 2021). If species hybridize in sympatry, we expect that individuals sampled in Santo Antonio estuary will be assigned to more than one cluster.

#### Phylogenetic relationships

We evaluated phylogenetic relationships among species based on our genomic data, both using phylogenomic and population genomic methods, that differ in their assumptions. First, we built a Maximum Likelihood (ML) phylogenetic tree describing the relationships among all individuals, classified according to morphology. We used dartR to produce a FASTA alignment for each individual containing a concatenation of all loci, with a random allele in heterozygous sites, using the six species datasets (6sp_0MD, 6sp_20MD, and 6sp_40MD). We used RAxML v. 8 (Stamatakis 2014) through CIPRES gateway (Miller et al. 2010) to perform 1 k bootstrap replicates (bs), with the GTR + GAMMA model. We visualized the ML tree using FigTree v.1.4.4 (http://tree.bio.ed.ac.uk/software/figtree/), rooting the tree with *M. liza* (Neves et al. 2020). We expect individuals of the same species to form monophyletic clades, either reflecting their mitochondrial or morphological classification.

Second, we built a species tree describing the relationships between the species included in our sampling while allowing for interspecific gene flow using TreeMix (Pickrell and Pritchard 2012). We used the most stringent dataset (6sp_0MD) to generate a NEXUS alignment where individuals were grouped a priori according to the five genetic clusters supported by mitochondrial and nuclear data (Fig. 2). We ran this analysis considering zero to three migration axes between species, with five iterations for each, recording the likelihood of each model and the *p*-value of each migration event.

#### Genetic differentiation and variability

We estimated genetic variability between and within species. To ensure an unbiased comparison among species, we used the comparative dataset without missing data (6sp_0MD). First, we estimated genetic differentiation between all pairwise comparisons, using the fixation index F_ST_ (Wright 1943), as implemented in StAMPP package (Pembleton et al. 2013). We grouped the individuals into the five nuclear genetic groups, but we kept the individuals morphologically identified as *M. incilis* separate and considered the two sampling locations of *M. curvidens* as distinct populations, to test for population differentiation. Second, we estimated several indices of genetic diversity within each of the five species supported by mitochondrial and nuclear data (Fig. 2) as a proxy for relative differences in effective population size (Ne). Using the HIERFSTAT package (Goudet 2005) and the dataset with 0% missing data (6sp_0MD), we estimated expected heterozygosity (He), observed heterozygosity (Ho), and inbreeding coefficient (F_IS_) (Wright 1943). We estimated 95% confidence intervals for F_IS_ by performing 100 replicates of non-parametric bootstrapping. Allele richness was estimated using the package PopGenReport (Adamack and Gruber 2014). Using the same dataset but concatenating the whole fragment instead, and using two haplotypes per individual without ambiguity codes, we estimated nucleotide diversity (*θ* and *π*), number of singletons, and departures from demographic stability with Tajima’s D, using DNAsp software v.6.12.03 (Rozas et al. 2017).

#### Demographic history

From a set of candidate models, we inferred the demographic history that best explains the observed patterns of genetic diversity within each species using the diffusion approximation methods implemented in δaδi (Gutenkunst et al. 2009). We produced one-dimensional site-frequency spectrum (SFS) for each species using the 40MD species-specific datasets after conversion to VCF using the radiator package (Gosselin 2017). To maximize the number of segregating sites in each SFS, we projected down to 80% the actual sample size, which uses subsampling to incorporate positions with missing data. This maximized the number of segregating sites in the SFS for most species when compared to projections to 100% or 60% the actual sample size. We folded and fit these SFSs to four models of increasing demographic complexity: (1) a *neutral* model, assuming a constant population size; (2) a *two-epoch* model, describing an instantaneous change in population size (*Ne*1) at a certain time (*T*1); (3) a *bottlegrowth* model, describing an instantaneous size change similar to the previous one (*Ne*1) at a certain time (*T*1), but followed by a period of exponential size change to the present size (*Ne*2); and (4) a *three-epoch* model, describing two instantaneous size changes (*Ne*1, *Ne*2) at times *T*1 and *T*2. We performed optimization using the δaδi pipeline developed by Portik et al. (2017), which performs multiple rounds of optimization, each round using the best fitting parameters from the last as new starting parameters. We performed these optimizations under default settings, with the following exceptions: we performed four rounds of optimization with [10,10,10,5] replicates in each round with maximum iterations of [5,30,60,700] per replicate in each round; we set a maximum limit of 50 on population size parameters; we optimized using the L-BFGS-B method. We replicated this approach three times per species and model combination to ensure convergence on model selection and parameter estimates. We plotted the optimized model SFS against the data SFS along with the residuals to infer deviations of the empirical data relative to the optimized model. To select the most likely demographic model for each species while accounting for the different number of parameters of the four models, we used the Akaike information criterion (AIC) (Akaike 1974), calculating AIC weights for each model and species combination (Burnham and Anderson 2002). Standard deviation for each parameter was calculated through the Fisher information matrix uncertainty estimation implemented in δaδi (Coffman et al. 2016).

## Results

### Data filtering

The two pairs of enzymes showed equivalent genetic distances between species, but *Pst*I-*Shp*I showed higher reproducibility between technical replicates (Table S2) and therefore was used to genotype all 94 individuals.

Our raw data (Fig. S2) was composed of 55,507 loci of ~69 bp with 71,585 SNPs. We observed a large amount of missing data (53%) that is not homogeneously distributed across species. By plotting the call rate of all loci by species (Fig. S3) we consistently observed bi-modal distributions, showing that loci are either always or never called across individuals of the same species. The most divergent species, *Mugil liza*, shows the largest amount of missing data (85%), followed by *M. curema* (72%). This is consistent with the absence of homologous restriction sites between highly divergent genomes, rather than a methodological error.

Considering all individuals together (i.e., in the 6sp datasets), we found 7,495 SNPs with 40% MD, 3,445 SNPs with 20% MD and 984 SNPs with 0% MD (Fig. S4). By removing individuals of *Mugil liza* (i.e., in the 5sp_0MD), the number of SNPs increases nearly two-fold (1,879 SNPs). When considering each individual assigned to each of the five mitochondrial clades separately, the number of SNPs is relatively low, considering 40% of missing data: 706 SNPs for *M. liza*, 946 for *M. brevirostris*, 2,001 for *M. rubrioculus*, 1,211 for *M. curema*, and 5,899 for *M. curvidens*. *Mugil curvidens* dataset with no missing data consist of 3,312 SNPs (Table S3 and S4).

### Analyses

#### Population structure

The first four dimensions of the PCoA using the comparative dataset without missing data (6sp_0MD) explained 95.4% of the data variability (Fig. 2A). In general, individuals from the same morphological species clustered together, with individuals of *M. curvidens* sampled at the two localities clustering together. The individuals that are morphologically assigned to *M. incilis* (2) and to *M. rubrioculus* (4) but that are mitochondrially assigned to *M. curema* clustered with the remaining individuals of *M. curema*. The individual that is morphologically assigned to *M. curema* but that has the mitochondrial lineage of *M. curvidens* also clustered with the remaining individuals of *M. curvidens*. Allowing for missing data did not change these results (Fig. S5).

In agreement, our STRUCTURE analyses show that the sampled individuals are assigned to five well-differentiated clusters, with the highest likelihood values at *K* = 5 (Fig. 2B; Fig. S6). The nuclear clusters correspond perfectly to the five mitochondrial lineages, confirming the misidentifications based on morphology. The two populations of *M. curvidens* are assigned to the same cluster. Our results show no sign of ongoing hybridization, as every individual is almost entirely assigned to a single cluster (maximum fraction detected from another cluster is 0.045). These results remained constant when performing the same analysis without *M. liza* (1,879 SNPs, Fig. S7A; maximum fraction = 0.044) or when only considering *M. curvidens* (3,312 SNPs, Fig. S7B; no individual is assigned to the second cluster with a fraction above 0.566).

#### Phylogenetic relationships

**Fig. 3:**
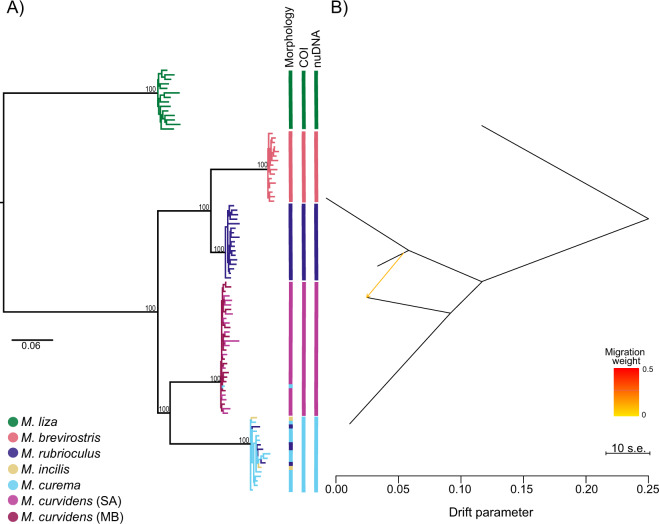
Phylogenetic history of *Mugil* species based on 984 SPs. **A** Maximum Likelihood phylogeny; numbers on nodes represent bootstrap support. Colors of the terminal branches refer to the morphological identification. **B** Species tree estimated by TreeMix showing interspecific gene flow between *M. rubrioculus* and *M. curvidens*.

Our ML tree (Fig. 3A) recovered five well supported clades (bs = 100), with a consistent topology to what was previously described using a fragment of the mitochondrial COI gene (Neves et al. 2021). The *M. curvidens* individuals sampled in the Manguaba and Santo Antonio estuaries form a single clade, suggesting no population-level divergence. Also in agreement with the mitochondrial DNA, the two individuals morphologically identified as *M. incilis* and the four individuals morphologically identified as *M. rubrioculus* nested within the clade of *M. curema*, being sister of *M. curvidens*, where the individual morphologically identified as *M. curema* nested. *Mugil brevirostris* is sister to *M. rubrioculus*, having the shortest branch lengths between species. The analysis including 20 and 40% of missing data showed the same topology (Fig. S8).

Our estimated species tree showed the same topology and relative branch lengths of the ML tree (Fig. 3B). The model assuming one migration was the simplest model describing the data, as more complex models did not retrieve significant migration events (Table S5). Thus, we only find significant admixture between *M. rubrioculus* and *M. curvidens* (*p*-value = 0.014).

#### Genetic differentiation and variability

Our measures of genetic differentiation between populations showed extremely high *F*_ST_ values between the five species identified by our population structure analyses (Table S6), showing that most SNPs are fixed between species. The minimum differentiation was observed between *M. brevirostris* and *M. rubrioculus* (*F*_ST_ = 0.851), and the maximum was between *M. liza* and *M. brevirostris* (*F*_ST_ = 0.948). Again, the individuals morphologically assigned to *M. incilis* but genetically identified as *M. curema* showed very low differentiation relative to *M. curema* (*F*_ST_ = 0.008). The two sampling localities of *M. curvidens* are also genetically similar (*F*_ST_ = 0.01). For most species (*M. brevirostris*, *M. rubrioculus*, and *M. curema*), the confidence intervals of *F*_IS_ included zero (Table 1), consistent with random mating. The remaining two species (*M. liza* and *M. cirvidens*) the confidence intervals of *F*_IS_ exclude zero, but yet overlap with the remaining species, suggesting similar inbreeding coefficients.

**Table 1:** Summary statistics of *Mugil* lineages from Tropical Southwestern Atlantic marine province.

Species	*N*	Diversity indexes							Fixation index	Demographic indexes	
		*H*_o_	*H*_e_	*S*	*A*_R_	*θ*	*π*	*F*_IS_		Singletons	Tajima’s D
***M. liza***	14	0.1868	0.2028	143	1.1295	0.00058	0.00046	0.0788 (0.0233–0.1408)		57	−0.82961
***M. brevirostris***	16	0.1544	0.1645	83	1.0698	0.00032	0.00021	0.016 (−0.0077–0.1453)		41	−1.27468
***M. rubrioculus***	17	0.1423	0.1414	124	1.0999	0.00048	0.00028	−0.0067 (−0.056–0.0407)		68	−1.58918
***M. curema***	17	0.1392	0.1508	109	1.0882	0.00042	0.00026	0.0765 (−0.0033–0.1451)		59	−1.44588
***M. curvidens***	30	0.0846	0.0888	167	1.1020	0.00056	0.00023	0.0473 (0.0088–0.0887)		97	−2.06450^a^

*N* sample size; *H*_o_ observed heterozygosity; *H*_e_ expected heterozygosity; *S* number of polymorphic sites; *A*_R_ mean allele richness; *θ* nucleotide diversity per site in the sequences; *π* average number of nucleotide differences per site; *F*_IS_ individual fixation index with 95% confidence intervals;

^a^denotes statistically significant deviations from zero (*p* < 0.05).

Our estimated levels of genetic diversity within species based on the same SNPs across species (Table 1) showed that *Mugil liza* has the highest values across most diversity indexes (*H*_o_, *H*_e_, *A*_R_, *θ*, and *π*), with the exception of the number of polymorphic sites (*S*) that was highest in *M. curvidens*. The lowest diversity was found in *M. curvidens* when considering heterozygosity (*H*_o_ and *H*_e_), or in *M. brevirostris* when considering other diversity indexes (*S*, *A*_R_, *θ*, and *π*). All species showed a negative Tajima’s D, suggestive of demographic expansion. But Tajima’s D was only significant in *M. curvidens* (*p* < 0.05), which shows the highest number of singletons.

#### Demographic history

**Fig. 4:**
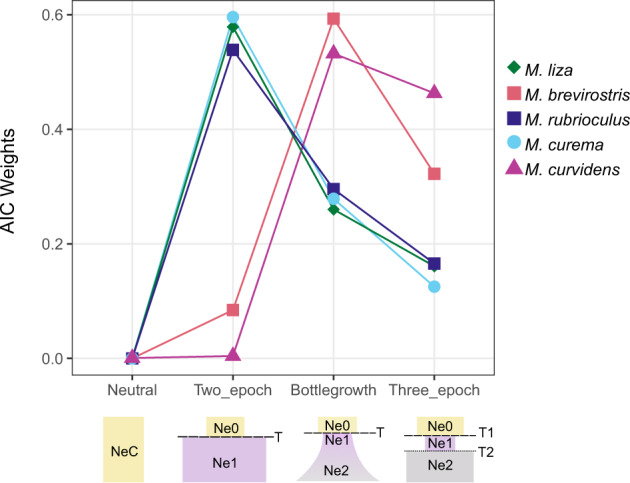
Demographic history of the five sympatric *Mugil* species. Weighted support for alternative demographic models according to Akaike’s information criterion (AIC).

Our demographic modeling rejected the *neutral* model of constant population size for all species, in favor of one of two similar models showing a recent range expansion: *two-epoch* and *bottlegrowth* (Fig. 4). When assuming a constant effective population size, our observed Site Frequency Spectra (SFS) showed an excess of singletons and a deficit of low-frequency SNPs across all species (Fig. S9). When including a change in effective population size, either instantaneous (*two-epoch*) or continuous (*bottlegrowth*) the SFS fit the expectations with few residuals (Fig. S10). The AIC weights (Fig. 4, Table S7) support *two-epoch* as the most probable of the candidate scenarios for *M. liza, M. rubrioculus*, and *M. curema* (AIC weights = 0.579, 0.539, and 0.596, respectively) and support *bottlegrowth* for *M. brevirostris* and *M. curvidens* (AIC weights = 0.593 and 0.532, respectively). In all cases, the AIC weight of the best fitting model is substantially higher than the second-best fitting, with the exception of *M. curvidens*, where *bottlegrowth* is only slightly more probable (AIC weight = 0.532) than *three-epoch* (AIC weight = 0.463), both estimating a similar scenario. The parameter estimates of the best fitting model describe a population expansion in all species, either directly from the ancestral population or after a bottleneck (Table S8). Parameter estimates from second-best models always show a similar result of population expansion (Table S9).

## Discussion

Designing sustainable and science-based measures for managing wild populations requires a fundamental knowledge on the number of species, their genetic connectivity, diversity, and demographic history (Scott et al. 2020; Bilgmann et al. 2021; Hoffmann et al. 2021). This is particularly challenging in morphologically similar species, such as the *Mugil* fishes, despite their high ecologic and economic importance. Here, we use a genomic approach to resolve these evolutionary questions and understand how speciation of these morphologically similar species can lead to the maintenance of strong genetic isolation in the absence of geographic isolation.

### Five species of *Mugil* occur in sympatry and show high vagility

The number of species of *Mugil* that are sympatric in the Coral Coast MPA in Brazil has been debated (Fig. 1) (Menezes et al. 2015; Barletta and Dantas 2016). A previous study integrating mitochondrial and morphological data reported a conflict between the two classifications (Table S1; Neves et al. 2021), suggesting either an overestimation of the number of species, or evolutionary processes that are specific to the mitochondrial genome.

Using hundreds to thousands of loci spread throughout the genome, our population structure analyses conclusively show that all sampled specimens belong to five well-defined genetic clusters (Fig. 2). These clusters align perfectly with the five mitochondrial lineages previously described based on the barcoding gene COI (Fig. 3A; Table S1; Neves et al. 2020). All individuals showing a conflict between the morphological and the mitochondrial classifications (two *M. incilis*, four *M. rubrioculus*, and one *M. curema*) were clustered with evolutionary lineages coincident with the mitochondrial gene, showing that this barcoding gene can reliably distinguish between species that cannot always be differentiated using external morphological data (Fig. 1). Although the number of genotyped individuals (94) and filtered SNPs used here (from 1,879 to 984 SNPs) are relatively modest, this result is consistent across datasets (Fig. S7A) and clustering methods (Fig. 2), showing that this sampling is sufficient to answer this research question, as shown in other RAD-seq studies with a smaller number of loci (Mesak et al. 2014). These results rule out the hypothesis of mitochondrial introgression through hybridization, and reduce the number of species in the study area to five: *M. liza*, *M. brevirostris*, *M. rubrioculus*, *M. curema*, and *M. curvidens*. Given that we find unexpectedly low level of homology between restriction sites across these five species (Figs. S2, S3), consistent with high genomic divergence in the nuclear genome (Table S6), future genomic studies would benefit from other next-generation sequencing methods that are more suitable for deeper time scales (da Fonseca et al. 2016).

Our results also show no significant genetic differentiation between the *M. curvidens* sampled in the two estuaries 38 km apart, even when this species was analyzed separately with double the number of loci (Fig. S7B). This confirms that the heterogenous habitat composed of coral reefs and recurrent plumes of sediments from the rivers (Passos et al. 2016) does not restrict gene flow and that these two areas with different conservation regimes harbor a single panmictic population of *M. curvidens*. Although this result is perhaps not surprising because adults of *Mugil* species migrate long distances along the shoreline (Livi et al. 2011) and because dispersal also occurs passively through pelagic eggs and larvae (Livi et al. 2011; Whitfield et al. 2012), this hypothesis had not been tested at the genomic level in *Mugil*. Such a level of genetic connectivity across heterogeneous habitats contrasts with genomic studies in other marine species (Riginos and Nachman 2001; Hauser and Carvalho 2008; Selkoe et al. 2016) and has relevant implications for conservation, as discussed below.

### *Mugil* species show strong genetic isolation despite full sympatry

Because many morphologically cryptic species are allopatric, showing a fractal genetic structure (Wake 2009; McMahan et al. 2013; Boehm et al. 2013), it is challenging to understand whether such speciation processes lead to strong reproductive isolation. In this context, sympatric cryptic species are important because we have an opportunity to measure genetic barriers between them and thus infer whether they represent evolutionarily stable units. According to a mitochondrial study (Neves et al. 2020), these focal species of *Mugil* have diverged between ~29 mya, when *M. liza* split from the remaining species, to ~6 mya, when *M. brevirostris* and *M. rubrioculus* diverged. Despite such a long period since initial divergence, it has not been tested whether these species were formed in the face of gene flow, and whether they currently present strong genetic isolation where they occur sympatrically, such as in this MPA.

Our maximum likelihood phylogenetic analysis using concatenated loci (Fig. 3A) estimates a topology and branch lengths that are in large agreement with those previously estimated from mitochondrial loci (Neves et al. 2020), irrespective of the missing data allowed (Fig. S8). This tree shows that *M. brevirostris* and *M. rubrioculus* diverged most recently, preceded by the split between *M. curema* and *M. curvidens*. Interestingly, most SNPs are fixed among all species pairwise comparisons, reflected in the extremely high values of fixation indices among the five species (Table S6) and consistent with old divergence.

By using allelic frequencies of independent SNPs for estimating phylogenetic relationships in the presence of incomplete lineage sorting and interspecific gene flow (Pickrell and Pritchard 2012), we find strong support for a topology that is congruent with the tree based on concatenated data. Notably, the most likely species tree shows gene flow between *M. rubrioculus* and *M. curvidens* (*p*-value = 0.014), without support for gene flow between all other species pairs (*p*-values > 0.278; Table S5). This suggests that, while some morphologically similar species might have formed in parapatry or sympatry, most species were likely formed in allopatry. This hypothesis is consistent with the extensive changes in the coast of South America that occurred from the Miocene (Bush, de Oliveira 2006) to the Pleistocene (Ludt and Rocha 2015), including a reduction of habitable area, changes in ocean currents, and shifts in water column thermal dynamics. Such changes have been associated to population bottlenecks in glacial refugia, and species formation in coastal marine taxa from tropical waters (Ludt and Rocha 2015), consistent with our findings for *Mugil*.

Our STRUCTURE analyses show that every individual has ancestry in a single cluster (membership probabilities > 0.95), irrespective of the stringency of the filtering (Fig. 2B, Fig. S7A), suggesting strong genetic isolation between species. This absence of hybrids suggests that genetic, behavioral, ecological, or other barriers can establish strong genetic barriers between species, despite morphological and spatial overlap. Although currently these species lack any geographic barriers, use the same macrohabitat, and are morphologically similar, several studies of *Mugil* species have shed some light on multiple reproductive barriers that might contribute to the strong reproductive isolation reported here. Ethological studies have shown that the spawning season of *M. curema* and *M. liza* only overlaps in one month in southeastern Brazil (Albieri et al. 2010), while the spawning season of *M. cephalus* and *M. curema* do not overlap in Mexico (Ibáñez-Aguirre 1993), suggesting that temporal isolation might have been established between several *Mugil* species. Ecological studies have found that in southeastern Brazil, *M. curema* is associated with high salinity waters while *M. liza* is associated with lower salinity (Mai et al. 2018), suggesting some ecological isolation between these species. Diet studies have shown that sympatric species of *Mugil* in West Africa can present differential particle size selection to avoid competition (LeLoc’h et al. 2015), possibly constituting a further ecological isolating barrier. Furthermore, cytogenetic studies have shown that some of these species differ in the number and arrangement of chromosomes (Rossi et al. 2005; Harrison et al. 2007; Nirchio et al. 2017), possibly constituting a genetic isolating barrier. Although the relative contribution of these barriers has not been tested by experimental crosses, our observation of lack of hybrids in an area of sympatry between five different species of *Mugil* suggest that these species show strong reproductive isolation, and thus have passed the “grey zone of speciation” (sensus Roux et al. 2016). Most species past this level of divergence have accumulated strong ecomorphological differentiation. Our results add to evidence from other studies of morphologically similar species (e.g. Roux et al. 2013) that suggest that cryptic speciation can also result in strong genetic barriers between sympatric taxa.

### Species differ in their relative abundance and demographic expansion

Information regarding population size change is fundamental for understanding the evolutionary history of a species and to delineate conservation strategies (Ramakrishnan et al. 2005; Dussex and Robertson 2018). Yet, inferring the evolutionary processes underlying the patterns of genetic variation within species requires hundreds or thousands of genetic markers sampled randomly across the genome.

Using genomic data, we show that the diversity indexes vary strongly between species (Table 1). Because in an idealized Wright–Fisher population the nucleotide diversity *π* = 4 *N*_e_ *µ* (where *µ* is the mutation rate per nucleotide site per generation and *N*_e_ is the effective population size), our estimates of *π* should be proportional to differences in *N*_e_ between species when using the same loci across species (dataset 6sp_0MD). Although we find relatively higher diversity estimates (*H*_o_, *H*_e_, *θ*, and *π*) in *M. liza* and lower in *M. brevirostris*, (*H*_o_, *H*_e_, and *π*), caution is needed when interpreting *N*_e_, since these are estimates of long-term effective population size and may not scale linearly with contemporary census sizes (Braude and Templeton 2009; Leffler et al. 2012). For example, *M. liza* is characterized by a larger body size, has higher commercial value, and their census size has decreased in recent years (Mendonça and Bonfante 2011; Sant’Ana et al. 2017; Vieira et al. 2019). Therefore, current patterns of genetic diversity within *Mugil* species are likely to be affected by the demographic history of each species, rather than reflecting their current relative abundance.

Our demographic modeling (Fig. 4) clarifies how the demographic history of each species shapes the current estimates of *N*_e_. The *neutral* demographic scenario was rejected for every species (AIC weight < 0.001 for all species) in favor of scenarios with an increase of *N*_e_ over time; a sudden increase of *N*_e_ for *M. liza*, *M. curema*, and *M. rubrioculus*, and an exponential increase of *N*_e_ after a sudden bottleneck for *M. brevirostris* and *M. curvidens*. The simulated patterns of variability are very similar under the three candidate models of range expansion (Fig. S9) and thus caution is needed in trying to distinguish between them. AIC weights convey a conditional probability for each model in the pool, and while the best fitting models were nearly double the weight of the second-best fitting model in all but one taxa (*M. curvidens*), this weight never exceeded 0.6. However, even if we examine the second-best fitting models for each taxa, the selected models and their parameter estimates agree with the results of the best-fitting models (Table S9). These results are also consistent with our observation of negative Tajima’s D across species, even though they were only statistically significant in one (Table 1). Based on the *N*_e_ estimates for the most likely model (Table S8), we estimate around a threefold increase with no bottleneck in *N*_e_ in *M. liza*, *M. curema*, and *M. rubrioculus*, which shows the three highest *π* estimates. Both species with the lowest values of *π*—*M. brevirostris* and *M. curvidens*—show expansions after a bottleneck, with the bottleneck and expansion both being stronger in *M. brevirostris* than in *M. curvidens*. This suggests that the current *N*_e_ is strongly shaped by species-specific demographic history. However, it should be noted that these parameter estimates are not a perfect comparison between species as they are estimates contingent upon the model used, which may not reflect the true demographic history, only the most probable of our candidates and given our data. Due to high differences in AIC weight for models between species (Table S7), no one model could be fixed to all for estimating comparable magnitudes for size changes in these species.

Genomic studies in other exploited fish species have consistently found signatures of demographic expansion in the Atlantic herring (Barrio et al. 2016), the North American lake whitefish (Rougeux et al. 2017), turbot (Momigliano et al. 2020), and Pacific salmon (Rougemont et al. 2020). Our results are in line with those findings and suggest that current patterns of genetic diversity in wild populations are highly determined by historical changes in *N*_e_ associated with the Quaternary ice age. Thus, future studies in protected areas must consider the effect of these important historical events shaping current patterns of genetic diversity. Higher *N*_e_ values can also be associated with maturation time lengthened (Nunney 1993), which can happen when juveniles maturate earlier due to overexploitation (Kuparinen and Merilä 2007), or with differences in geographical range among species (Leffler et al. 2012). This suggest that, although these species are morphologically similar and coexist in the same habitats, their evolutionary histories can be quite different, affecting their levels of standing genetic variation, and hence their adaptive potential (Wang et al. 2016).

### Implications for conservation

Although governmental institutions and legislators are willing to implement new science-based regulations, a fundamental knowledge on the number of species, their genetic connectivity, and their demographic history is still lacking for most species of ecological and economic interest. Considering that Mugilidae fishes are particularly challenging to identify morphologically, yet are the main fish target in tropical artisanal fisheries (Batista et al. 2014), our results provide important guidelines for conservation.

The data generated by the DArT-seq method can provide valuable information for conservation and management, having been applied to species of great economic importance that have been historically exploited. Some examples are the lobster *Panulirus homarus* (Palinuridae) exploited in the Middle East (Al-Breiki et al. 2018), the sharks *Carcharhinus brachyurus* and *C. obscurus* (Carcharhinidae) (Junge et al. 2019), shrimps of the genus *Macrobrachium* (Palaemonidae) (Makombu et al. 2019) exploited globally, and fish species of the genus *Osteoglossum* (Osteoglossidae) commercialized in the Amazon region (de Souza et al. 2019).

We show that *M. incilis* is very likely absent from the Coral Coast MPA, in agreement with a previous study based on morphological and mitochondrial data (Menezes et al. 2015). This implies that conservation efforts inside this MPA should focus only on five *Mugil* species and that *M. incilis* has a more restricted distribution than previously thought.

Currently, fisheries in northeastern Brazil report capture of all *Mugil* species under the same category (9,219.5 tons in 2007; IBAMA 2007). Irrespective of their unknown current census population sizes, our results suggest that these species have different effective population sizes (Table 1), and that the magnitude and mode of demographic expansion differs across species (Table S8). Although our results suggest that each species of *Mugil* should have a specific protection status, this is extremely challenging to implement in such a morphologically conserved group of species. Yet, our finding that species classification using thousands of genomic markers is in strict agreement with species classification based on a single fragment of the COI gene (Neves et al. 2021) represents an important validation for future monitorization studies using hundreds or thousands of samples. This mitochondrial barcoding gene provides a cost-effective tool to monitor fisheries bycatch, and thus to assess species abundance over time and to establish sustainable protective measure for such ecologically and economically important species.

## Data archiving

All individuals sampled for this study were deposited in the ichthyology collection of the Alagoas university; voucher number, sampling locations, morphological data and COI Genbank accession numbers are listed in Table S1. All the raw genomic data, filtered datasets, scripts and infiles for all the analyses were deposited in GitHub (https://github.com/JMNeves/mugil_dart).

## Supplementary information

Supplementary Figures

Supplementary Tables

## References

[CR1] Adamack AT, Gruber B (2014). PopGenReport: simplifying basic population genetic analyses in R. Methods Ecol Evol.

[CR2] Akaike H (1974). A new look at the statistical model identification. IEEE Trans Autom Contr.

[CR3] Al-Breiki RD, Kjeldsen SR, Afzal H, Al Hinai MS, Zenger KR, Jerry DR (2018). Genome-wide SNP analyses reveal high gene flow and signatures of local adaptation among the scalloped spiny lobster (*Panulirus homarus*) along the Omani coastline. BMC Genomics.

[CR4] Albieri RJ, Araújo FG, Uehara W (2010). Differences in reproductive strategies between two co-occurring mullets *Mugil curema* Valenciennes 1836 and *Mugil liza* Valenciennes 1836 (Mugilidae) in a tropical bay. Trop Zool.

[CR5] Allendorf FW (2017). Genetics and the conservation of natural populations: allozymes to genomes. Mol Ecol.

[CR6] Asgharian H, Sahafi HH, Ardalan AA, Shekarriz S, Elahi E (2011). Cytochrome c oxidase subunit 1 barcode data of fish of the Nayband National Park in the Persian Gulf and analysis using meta-data flag several cryptic species. Mol Ecol Resour.

[CR7] Barletta M, Dantas D (2016). Biogeography and distribution of Mugilidae in the Americas. In: Crosseti D, Blaber SJM (eds) Biology, ecology and culture of grey mullets (Mugilidae), CRC Press: Boca Raton, FL, pp 42–62.

[CR8] Barrio AM, Lamichhaney S, Fan G, Rafati N, Pettersson M, Zhang H (2016). The genetic basis for ecological adaptation of the Atlantic herring revealed by genome sequencing. Elife.

[CR9] Batista VS, Fabré NN, Malhado ACM, Ladle RJ (2014). Tropical artisanal coastal fisheries: challenges and future directions. Rev Fish Sci Aquac.

[CR10] Benzaquem DC, Oliveira C, Da Silva Batista J, Zuanon J, Porto JIR (2015). DNA barcoding in pencilfishes (Lebiasinidae: Nannostomus) reveals cryptic diversity across the Brazilian Amazon. PLoS ONE.

[CR11] Bilgmann K, Armansin N, Ferchaud AL, Normandeau E, Bernatchez L, Harcourt R, et al. (2021). Low effective population size in the genetically bottlenecked Australian sea lion is insufficient to maintain genetic variation. Anim Conserv. 10.1111/acv.12688

[CR12] Boehm JT, Woodall L, Teske PR, Lourie SA, Baldwin C, Waldman J, et al. (2013). Marine dispersal and barriers drive Atlantic seahorse diversification (L Rocha, Ed.). J Biogeogr: n/a-n/a.

[CR13] Brandão JHSG, Bitencourt J, de A, Santos FB, Watanabe LA, Schneider H, Sampaio I (2016). DNA barcoding of coastal ichthyofauna from Bahia, northeastern Brazil, South Atlantic: high efficiency for systematics and identification of cryptic diversity. Biochem Syst Ecol.

[CR14] Braude S, Templeton AR (2009). Understanding the multiple meanings of ‘inbreeding’ and ‘effective size’ for genetic management of African rhinoceros populations. Afr J Ecol.

[CR15] Burnham KP, Anderson DR (2002). Model selection and multimodel inference. A practical information-teoretic approach.

[CR16] Bush MB, de Oliveira PE (2006). The rise and fall of the refugial hypothesis of Amazonian speciation: a paleoecological perspective. Biota Neotrop 6

[CR17] Carnaval AC, Hickerson MJ, Haddad CFB, Rodrigues MT, Moritz CC (2009). Stability predicts genetic diversity in the Brazilian atlantic forest hotspot. Science.

[CR18] Coffman AJ, Hsieh PH, Gravel S, Gutenkunst RN (2016). Computationally efficient composite likelihood statistics for demographic inference. Mol Biol Evol.

[CR19] Cook CN, Sgrò CM (2019). Poor understanding of evolutionary theory is a barrier to effective conservation management. Conserv Lett.

[CR20] Crandall KA, Bininda-Emonds ORR, Mace GM, Wayne RK (2000). Considering evolutionary processes in conservation biology. Trends Ecol Evol.

[CR21] Crosetti D (2016). Current state of grey mullet fisheries and culture. In: Crosseti D, Blaber SJM (eds) Biology, ecology and culture of grey mullets (Mugilidae), CRC Press: Boca Raton, FL, pp 398–450.

[CR22] da Fonseca RR, Albrechtsen A, Themudo GE, Ramos-Madrigal J, Sibbesen JA, Maretty L (2016). Next-generation biology: sequencing and data analysis approaches for non-model organisms. Mar Genomics.

[CR23] de Souza CD, Batista V, da S, Fabré NN (2012). Caracterização da pesca no extremo sul da área de proteção ambiental Costa dos Corais, Alagoas, Brasil. Bol do Inst Pesca.

[CR24] de Souza FHS, Perez MF, Bertollo LAC, de Oliveira EA, Lavoué S, Gestich CC (2019). Interspecific genetic differences and historical demography in South American arowanas (Osteoglossiformes, Osteoglossidae, Osteoglossum). Genes.

[CR25] Delrieu-Trottin E, Durand J, Limmon G, Sukmono T, Kadarusman K, Sugeha HY (2020). Biodiversity inventory of the grey mullets (Actinopterygii: Mugilidae) of the Indo-Australian Archipelago through the iterative use of DNA-based species delimitation and specimen assignment methods. Evol Appl.

[CR26] Durand JD, Borsa P (2015). Mitochondrial phylogeny of grey mullets (Acanthopterygii: Mugilidae) suggests high proportion of cryptic species. Comptes Rendus.

[CR27] Durand JD, Chen WJ, Shen KN, Fu C, Borsa P (2012). Genus-level taxonomic changes implied by the mitochondrial phylogeny of grey mullets (Teleostei: Mugilidae). Comptes Rendus.

[CR28] Durand JD, Shen KN, Chen WJ, Jamandre BW, Blel H, Diop K (2012). Systematics of the grey mullets (Teleostei: Mugiliformes: Mugilidae): Molecular phylogenetic evidence challenges two centuries of morphology-based taxonomy. Mol Phylogenet Evol.

[CR29] Durand JD, Whitfield AK (2016). Biogeography and distribution of Mugilidae in the western, central and southern regions of Africa. In: Crosseti D, Blaber SJM (eds) Biology, ecology and culture of grey mullets (Mugilidae), CRC Press: Boca Raton, FL, pp 102–115.

[CR30] Dussex N, Robertson BC (2018). Contemporary effective population size and predicted maintenance of genetic diversity in the endangered kea (*Nestor notabilis*). N. Zeal J Zool.

[CR31] Galetti PM, Aguilar CT, Molina WF (2000). An overview of marine fish cytogenetics. Hydrobiologia.

[CR32] Galtier N, Nabholz B, Glémin S, Hurst GDD (2009). Mitochondrial DNA as a marker of molecular diversity: a reappraisal. Mol Ecol.

[CR33] Gosselin T (2017). Radiator: RADseq Data Exploration, Manipulation and Visualization Using R.

[CR34] Goudet J (2005). HIERFSTAT, a package for R to compute and test hierarchical F-statistics. Mol Ecol Notes.

[CR35] Grewe PM, Feutry P, Hill PL, Gunasekera RM, Schaefer KM, Itano DG (2015). Evidence of discrete yellowfin tuna (*Thunnus albacares*) populations demands rethink of management for this globally important resource. Sci Rep..

[CR36] Gruber B, Unmack PJ, Berry OF, Georges A (2018). dartr: an r package to facilitate analysis of SNP data generated from reduced representation genome sequencing. Mol Ecol Resour.

[CR37] Grundler MR, Singhal S, Cowan MA, Rabosky DL (2019). Is genomic diversity a useful proxy for census population size? Evidence from a species-rich community of desert lizards. Mol Ecol.

[CR38] Gutenkunst RN, Hernandez RD, Williamson SH, Bustamante CD (2009). Inferring the joint demographic history of multiple populations from multidimensional SNP frequency data. PLoS Genet.

[CR39] Harrison IJ, Nirchio M, Oliveira C, Ron E, Gaviria J (2007). A new species of mullet (Teleostei: Mugilidae) from Venezuela, with a discussion on the taxonomy of *Mugil gaimardianus*. J Fish Biol.

[CR40] Hauser L, Carvalho GR (2008). Paradigm shifts in marine fisheries genetics: ugly hypotheses slain by beautiful facts. Fish Fish.

[CR41] Hoffmann AA, Miller AD, Weeks AR (2021). Genetic mixing for population management: from genetic rescue to provenancing. Evol Appl.

[CR42] IBAMA (2007). Estatística da pesca 2007 Brasil: Grandes regiões e unidades da federação. Brasília

[CR43] Ibáñez-Aguirre AL (1993). Coexistence of *Mugil cephalus* and *M. curema* in a coastal lagoon in the Gulf of Mexico. J Fish Biol.

[CR44] Junge C, Donnellan SC, Huveneers C, Bradshaw CJA, Simon A, Drew M (2019). Comparative population genomics confirms little population structure in two commercially targeted carcharhinid sharks. Mar Biol.

[CR45] Kopelman NM, Mayzel J, Jakobsson M, Rosenberg NA, Mayrose I (2015). CLUMPAK: a program for identifying clustering modes and packaging population structure inferences across K. Mol Ecol Resour.

[CR46] Kuparinen A, Merilä J (2007). Detecting and managing fisheries-induced evolution. Trends Ecol Evol.

[CR47] Lebreton B, Richard P, Parlier EP, Guillou G, Blanchard GF (2011). Trophic ecology of mullets during their spring migration in a European saltmarsh: a stable isotope study. Estuar Coast Shelf Sci.

[CR48] Leffler EM, Bullaughey K, Matute DR, Meyer WK, Ségurel L, Venkat A (2012). Revisiting an old riddle: what determines genetic diversity levels within species?. PLoS Biol.

[CR49] LeLoc’h F, Durand JD, Diop K, Panfili J (2015). Spatio-temporal isotopic signatures (δ13C and δ15N) reveal that two sympatric West African mullet species do not feed on the same basal production sources. J Fish Biol.

[CR50] Livi S, Sola L, Crosetti D (2011). Phylogeographic relationships among worldwide populations of the cosmopolitan marine species, the striped gray mullet (*Mugil cephalus*), investigated by partial cytochrome b gene sequences. Biochem Syst Ecol.

[CR51] Ludt WB, Rocha LA (2015). Shifting seas: the impacts of Pleistocene sea-level fluctuations on the evolution of tropical marine taxa. J Biogeogr.

[CR52] Mai ACG, dos Santos ML, Lemos VM, Vieira JP (2018). Discrimination of habitat use between two sympatric species of mullets, *Mugil curema* and *Mugil liza* (Mugiliformes: Mugilidae) in the rio Tramandaí Estuary, determined by otolith chemistry. Neotrop Ichthyol.

[CR53] Makombu JG, Stomeo F, Oben PM, Tilly E, Stephen OO, Oben BO (2019). Morphological and molecular characterization of freshwater prawn of genus Macrobrachium in the coastal area of Cameroon. Ecol Evol.

[CR54] McBride CS, Van Velzen R, Larsen TB (2009). Allopatric origin of cryptic butterfly species that were discovered feeding on distinct host plants in sympatry. Mol Ecol.

[CR55] McMahan CD, Davis MP, Domínguez-Domínguez O, García-de-León FJ, Doadrio I, Piller KR (2013). From the mountains to the sea: phylogeography and cryptic diversity within the mountain mullet, *Agonostomus monticola* (Teleostei: Mugilidae). J Biogeogr.

[CR56] Mendonça J, Bonfante T (2011). Assessment and management of white mullet Mugil curema (Valencienne, 1836) (Mugilidae) fisheries of the south coast of São Paulo state, Brazil. Braz J Biol.

[CR57] Menezes NA, De Oliveira C, Siccha-Ramirez R (2015). Taxonomic review of the species of Mugil (Teleostei: Perciformes: Mugilidae) from the Atlantic South Caribbean and South America, with integration of morphological, cytogenetic and molecular data. Zootaxa.

[CR58] Mesak F, Tatarenkov A, Earley RL, Avise JC (2014). Hundreds of SNPs vs. dozens of SSRs: which dataset better characterizes natural clonal lineages in a self-fertilizing fish?. Front Ecol Evol.

[CR59] Miller MA, Pfeiffer W, Schwartz T (2010). Creating the CIPRES Science Gateway for inference of large phylogenetic trees. 2010 Gateway Computing Environments Workshop.

[CR60] Momigliano P, Florin A-B, Merilä J (2020). Biases in demographic modelling affect our understanding of recent divergence. bioRxiv.

[CR61] Mora C, Zapata FA (2013). Anthropogenic footprints on biodiversity. In: Rohde K (ed) The balance of nature and human impact, Cambridge University Press, pp 239–258.

[CR62] Moritz CC (2002). Strategies to protect biological diversity and the evolutionary processes that sustain it. Syst Biol.

[CR63] Moritz CC, Pratt RC, Bank S, Bourke G, Bragg JG, Doughty P (2018). Cryptic lineage diversity, body size divergence, and sympatry in a species complex of Australian lizards (Gehyra). Evolution.

[CR64] Neves JMM, Almeida JPFA, Sturaro MJ, Fabré NN, Pereira RJ, Mott T (2020). Deep genetic divergence and paraphyly in cryptic species of Mugil fishes (Actinopterygii: Mugilidae). Syst Biodivers.

[CR65] Neves JMM, Perez A, Fabré NN, Pereira RJ, Mott T (2021). Integrative taxonomy reveals extreme morphological conservatism in sympatric Mugil species from the Tropical Southwestern Atlantic. J Zool Syst Evol Res.

[CR66] Nirchio M, Cipriano R, Cestari M, Fenocchio A (2005). Cytogenetical and morphological features reveal significant differences among Venezuelan and Brazilian samples of Mugil curema (Teleostei: Mugilidae). Neotrop Ichthyol.

[CR67] Nirchio M, Oliveira C, Siccha-Ramirez ZR, de Sene VF, Sola L, Milana V (2017). The Mugil curema species complex (pisces, mugilidae): a new karyotype for the pacific white mullet mitochondrial lineage. Comp Cytogenet.

[CR68] Nordlie FG (2016). Adaptation to salinity and osmoregulation in Mugilidae. In: Crosseti D, Blaber SJM (eds) Biology, ecology and culture of grey mullets (Mugilidae), CRC Press: Boca Raton, FL, pp 293–323.

[CR69] Nunney L (1993). The influence of mating system and overlapping generations on effective population size. Evolution.

[CR70] Pacheco-Almanzar E, Ramírez-Saad H, Velázquez-Aragón JA, Serrato A, Ibáñez AL (2017). Diversity and genetic structure of white mullet populations in the Gulf of Mexico analyzed by microsatellite markers. Estuar Coast Shelf Sci.

[CR71] Pacheco-Almanzar E, Simons J, Espinosa-Pérez H, Chiappa-Carrara X, Ibáñez AL (2016). Can the name *Mugil cephalus* (Pisces: Mugilidae) be used for the species occurring in the north western Atlantic?. Zootaxa.

[CR72] Passos CVB, Fabré NN, Malhado ACM, Batista VS, Ladle RJ (2016). Estuarization increases functional diversity of demersal fish assemblages in tropical coastal ecosystems. J Fish Biol.

[CR73] Peart CR, Tusso S, Pophaly SD, Botero-Castro F, Wu CC, Aurioles-Gamboa D (2020). Determinants of genetic variation across eco-evolutionary scales in pinnipeds. Nat Ecol Evol.

[CR74] Pedersen CET, Albrechtsen A, Etter PD, Johnson EA, Orlando L, Chikhi L (2018). A southern African origin and cryptic structure in the highly mobile plains zebra. Nat Ecol Evol.

[CR75] Pembleton LW, Cogan NOI, Forster JW (2013). StAMPP: An R package for calculation of genetic differentiation and structure of mixed-ploidy level populations. Mol Ecol Resour.

[CR76] Peterson BK, Weber JN, Kay EH, Fisher HS, Hoekstra HE (2012). Double digest RADseq: an inexpensive method for de novo SNP discovery and genotyping in model and non-model species.. PLoS One.

[CR77] Pickrell JK, Pritchard JK (2012). Inference of population splits and mixtures from genome-wide allele frequency data. PLoS Genet.

[CR78] Portik DM, Leaché AD, Rivera D, Barej MF, Burger M, Hirschfeld M (2017). Evaluating mechanisms of diversification in a Guineo-Congolian tropical forest frog using demographic model selection. Mol Ecol.

[CR79] Pritchard JK, Stephens M, Donnelly P (2000). Inference of population structure using multilocus genotype data. Genetics.

[CR80] R Core Team (2020). R: a language and environment for statistical computing. R Foundation for Statistical Computing, Vienna, Austria.

[CR81] Ramakrishnan U, Hadly EA, Mountain JL (2005). Detecting past population bottlenecks using temporal genetic data. Mol Ecol.

[CR82] Riginos C, Nachman MW (2001). Population subdivision in marine environments: the contributions of biogeography, geographical distance and discontinuous habitat to genetic differentiation in a blennioid fish, *Axoclinus nigricaudus*. Mol Ecol.

[CR83] Rossi AR, Gornung E, Sola L, Nirchio M (2005). Comparative molecular cytogenetic analysis of two congeneric species, *Mugil curema* and *M. liza* (Pisces, Mugiliformes), characterized by significant karyotype diversity. Genetica.

[CR84] Rougemont Q, Moore JS, Leroy T, Normandeau E, Rondeau EB, Withler RE (2020). Demographic history shaped geographical patterns of deleterious mutation load in a broadly distributed Pacific Salmon. PLoS Genet.

[CR85] Rougeux C, Bernatchez L, Gagnaire PA (2017). Modeling the multiple facets of speciation-with-gene-flow toward inferring the divergence history of lake whitefish species pairs (*Coregonus clupeaformis*). Genome Biol Evol.

[CR86] Roux C, Fraïsse C, Romiguier J, Anciaux Y, Galtier N, Bierne N (2016). Shedding light on the grey zone of speciation along a continuum of genomic divergence. PLoS Biol.

[CR87] Roux C, Tsagkogeorga G, Bierne N, Galtier N (2013). Crossing the species barrier: genomic hotspots of introgression between two highly divergent *Ciona intestinalis* species. Mol Biol Evol.

[CR88] Rozas J, Ferrer-Mata A, Sanchez-DelBarrio JC, Guirao-Rico S, Librado P, Ramos-Onsins SE (2017). DnaSP 6: DNA sequence polymorphism analysis of large data sets. Mol Biol Evol.

[CR89] Sáez AG, Lozano E (2005). Body doubles. Nature.

[CR90] Sant’Ana R, Gerhard Kinas P, Villwock de Miranda L, Schwingel PR, Castello JP, Paes Vieira J (2017). Bayesian state-space models with multiple CPUE data: the case of a mullet fishery. Sci Mar.

[CR91] Scott PA, Allison LJ, Field KJ, Averill-Murray RC, Bradley Shaffer H (2020). Individual heterozygosity predicts translocation success in threatened desert tortoises. Science.

[CR92] Selkoe KA, D’Aloia CC, Crandall ED, Iacchei M, Liggins L, Puritz JB (2016). A decade of seascape genetics: contributions to basic and applied marine connectivity. Mar Ecol Prog Ser.

[CR93] Stamatakis A (2014). RAxML version 8: a tool for phylogenetic analysis and post-analysis of large phylogenies. Bioinformatics.

[CR94] Vieira J, Román-Robles V, Rodrigues F, Ramos L, dos Santos ML (2019). Long-term spatiotemporal variation in the juvenile fish assemblage of the tramandaí river estuary (29°S) and adjacent ocast in southern Brazil. Front Mar Sci 6.

[CR95] Wake DB (2009). What salamanders have taught us about evolution. Annu Rev Ecol Evol Syst.

[CR96] Wang J, Santiago E, Caballero A (2016). Prediction and estimation of effective population size. Heredity.

[CR97] Ward RD, Hanner R, Hebert PDN (2009). The campaign to DNA barcode all fishes, FISH-BOL. J Fish Biol.

[CR98] Whitfield AK, Panfili J, Durand JD (2012). A global review of the cosmopolitan flathead mullet *Mugil cephalus* Linnaeus 1758 (Teleostei: Mugilidae), with emphasis on the biology, genetics, ecology and fisheries aspects of this apparent species complex. Rev Fish Biol Fish.

[CR99] Wright S (1943). Isolation by distance. Genetics.

[CR100] WWF (2016). Living planet report 2016. Risk and resilience in a new era. Gland, Switzerland.

[CR101] Xia R, Durand JD, Fu C (2016). Multilocus resolution of Mugilidae phylogeny (Teleostei: Mugiliformes): implications for the family’s taxonomy. Mol Phylogenet Evol.

