# Supplementary material for: Genomic methods reveal independent demographic histories despite strong morphological conservatism in fish species
Source: Heredity (Edinb). 2021 Jul 5;127(3):323–33. doi: 10.1038/s41437-021-00455-4 (PMC8405619; doi:10.1038/s41437-021-00455-4)
Supplement: Supplementary file 1 — Supplementary Figures [file 41437_2021_455_MOESM1_ESM.pdf]

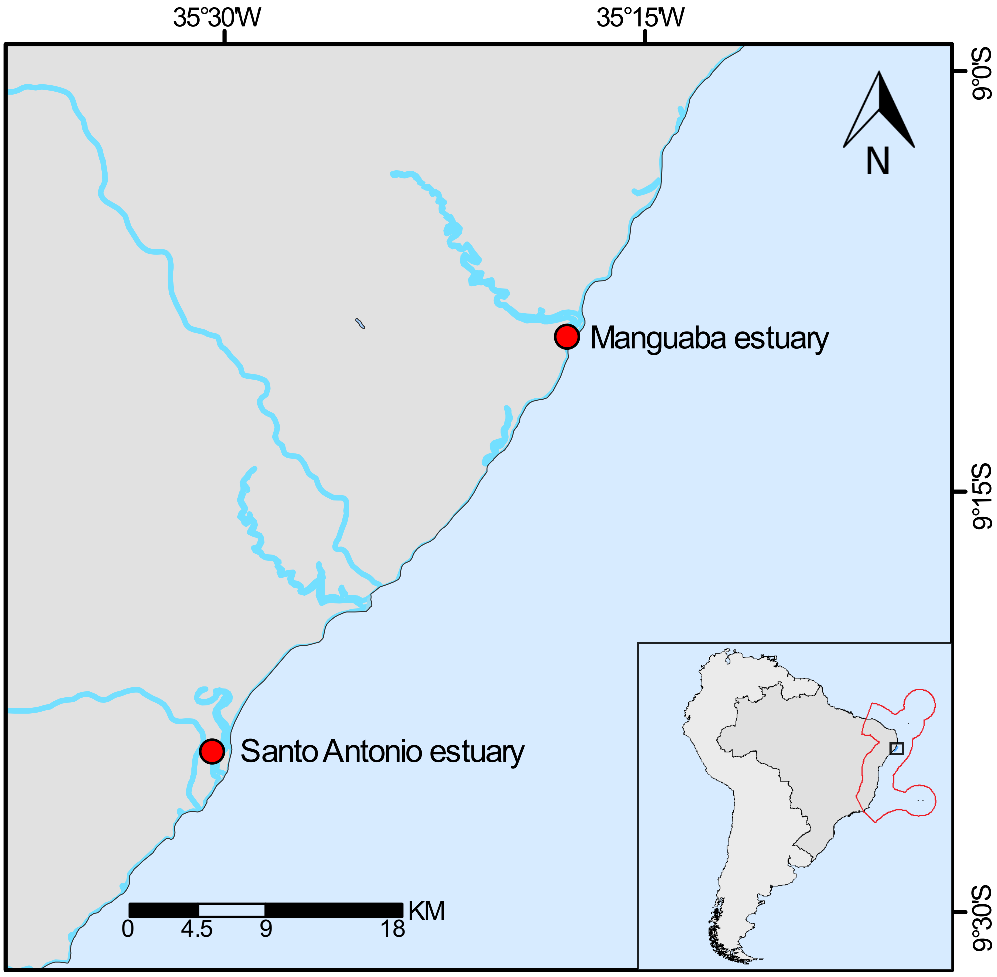

Figure S1. Sampling sites (red dots). The Tropical Southwestern Atlantic marine province is highlighted in inset map.

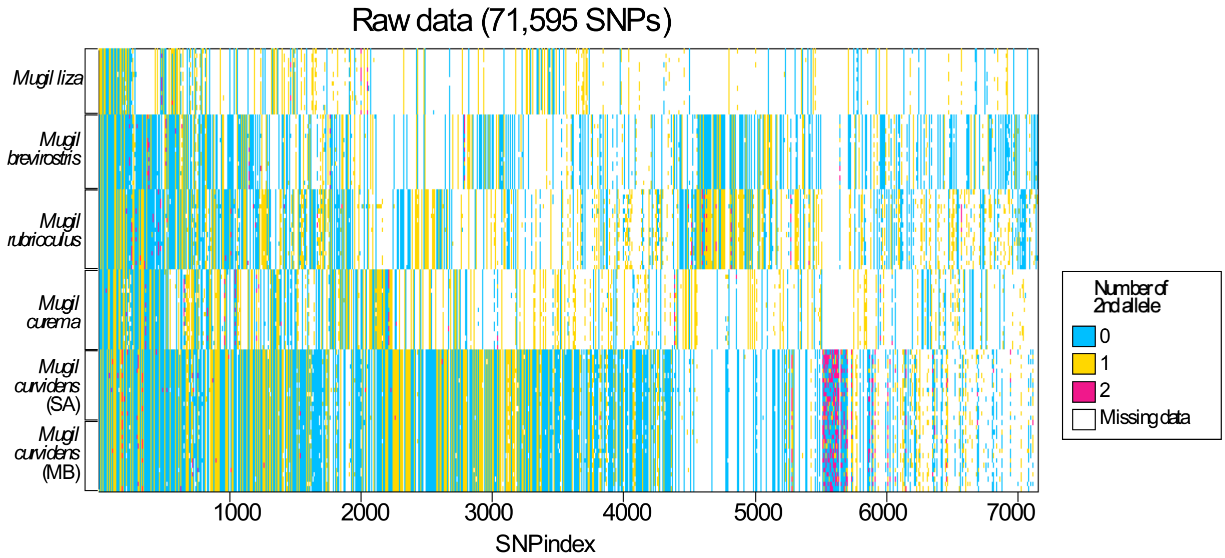

Figure S2. Raw data. Lines represent the 94 *Mugil* individuals, classified according to genetic clustering, and sampling locality (SA – individuals sampled at Santo Antonio estuary; MB – individuals sampled at Manguaba estuary). Columns represent the 71,595 SNPs before filtering.

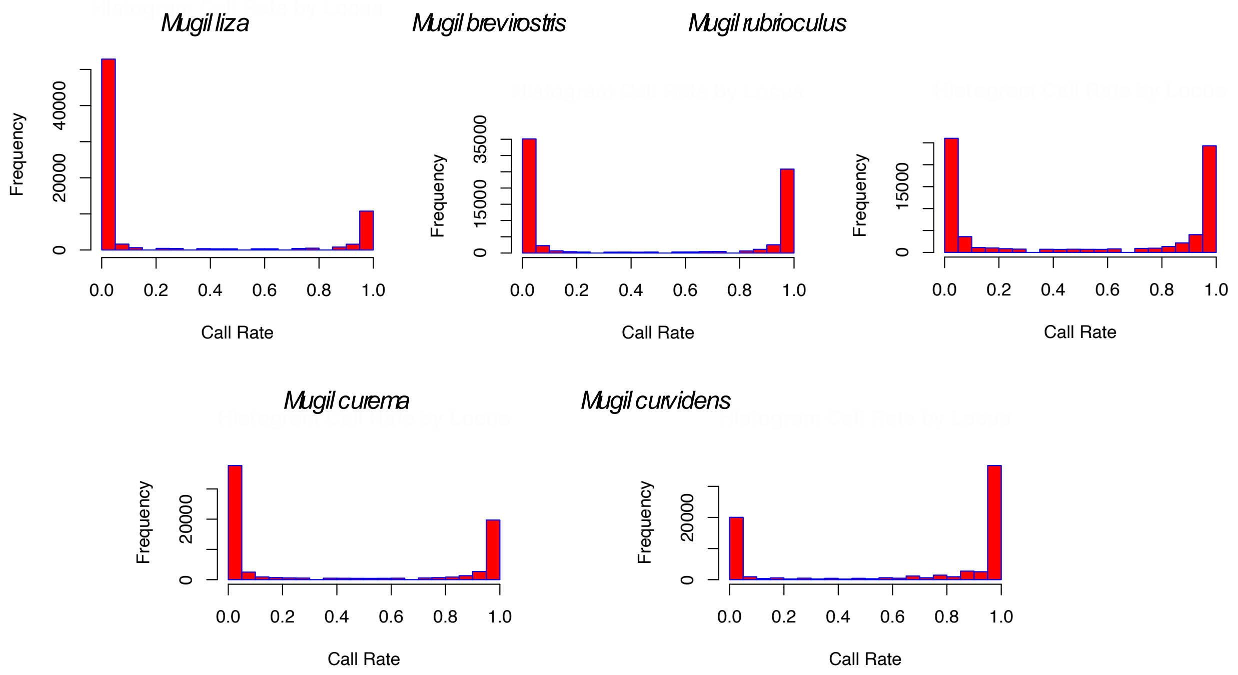

Figure S3. Histogram of the call rate of all loci in each *Mugil* species, classified according to the genetic clustering. The bi-modal distributions show that loci are either always or never called across all individuals of the same species.

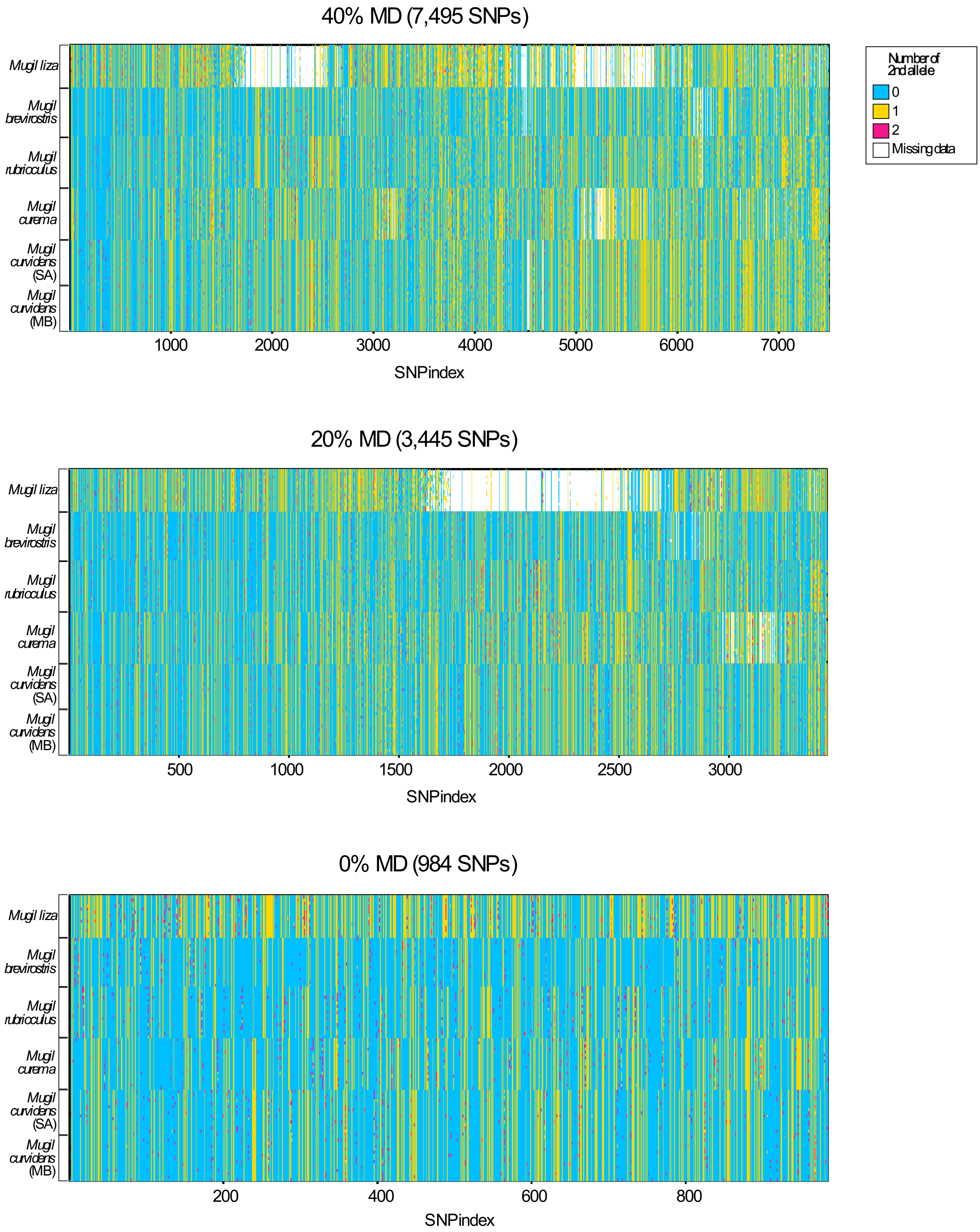

Figure S4. SNPs after filtering considering three levels of maximum missing data (MD) per locus. From the top to the bottom: 40% missing data resulted in 7,495 SNPs, 20% missing data resulted in 3,445 SNPs, and 0% missing data resulted in 984 SNPs. Lines represent the 94 *Mugil* individuals, classified according to genetic clustering and sampling localities (SA – individuals sampled at Santo Antonio estuary; MB – individuals sampled at Manguaba estuary).

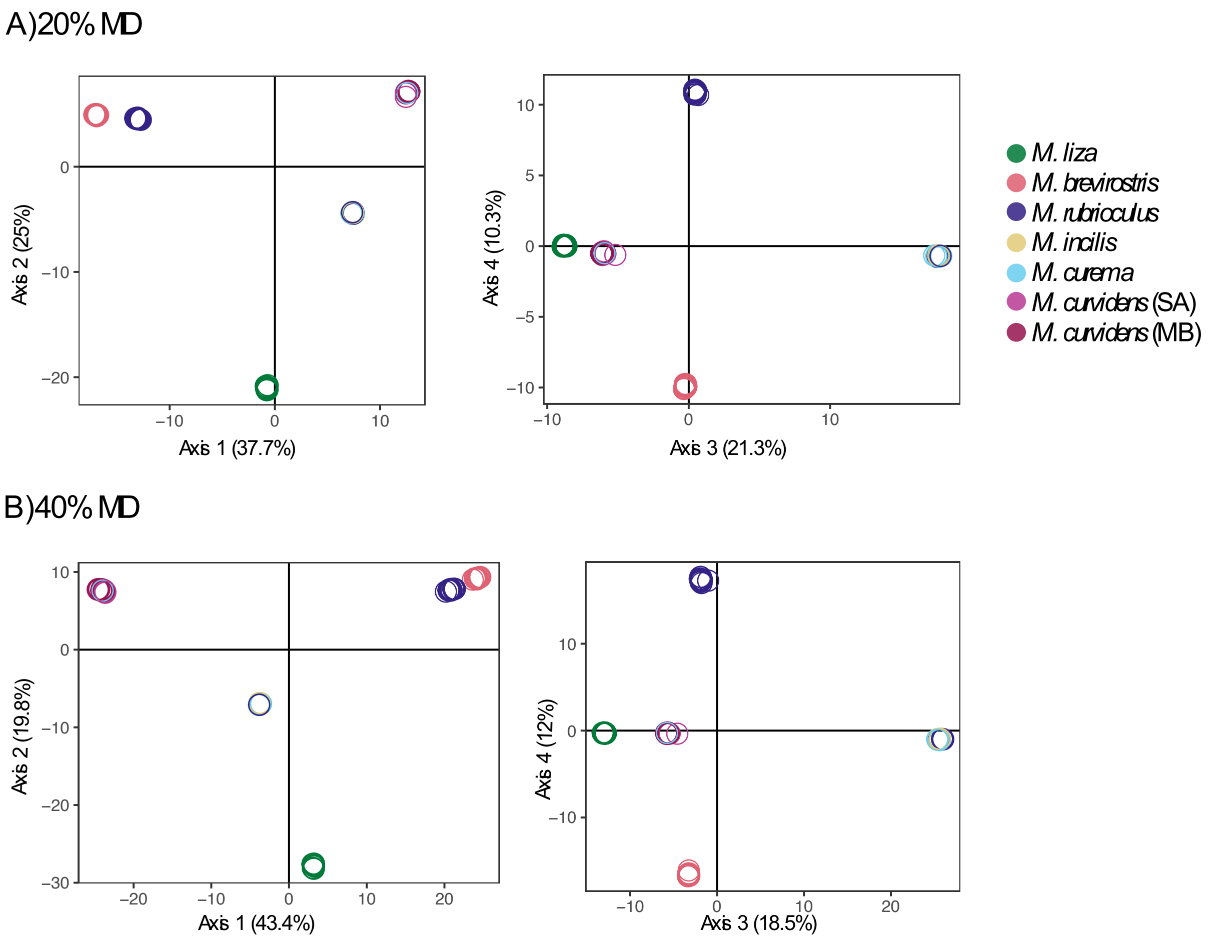

Figure S5. PCoA analysis considering a maximum of 20% (A) and 40% (B) of missing data with 94 *Mugil* individuals, classified according to morphological identification and sampling locality (SA – individuals sampled at Santo Antonio estuary; MB – individuals sampled at Manguaba estuary).

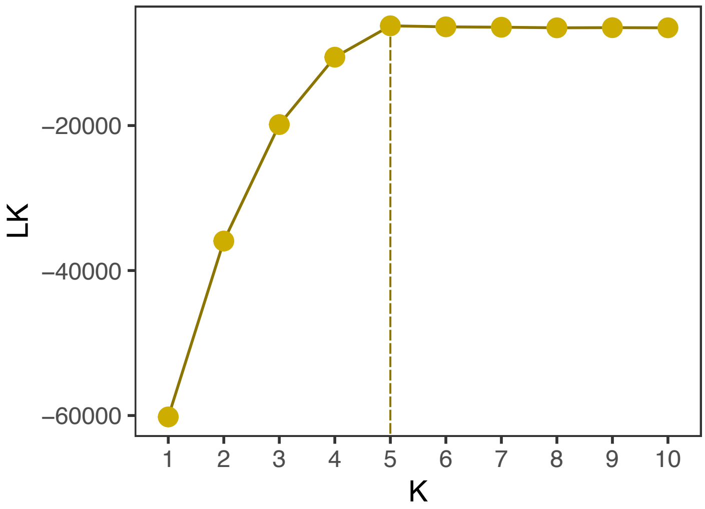

Figure S6. Log-likelihood plot of the STRUCTURE analysis using the dataset with 0% missing data (984 SNPs). The Log-likelihoods were highly consistent across all replicates, showing a peak at K=5.

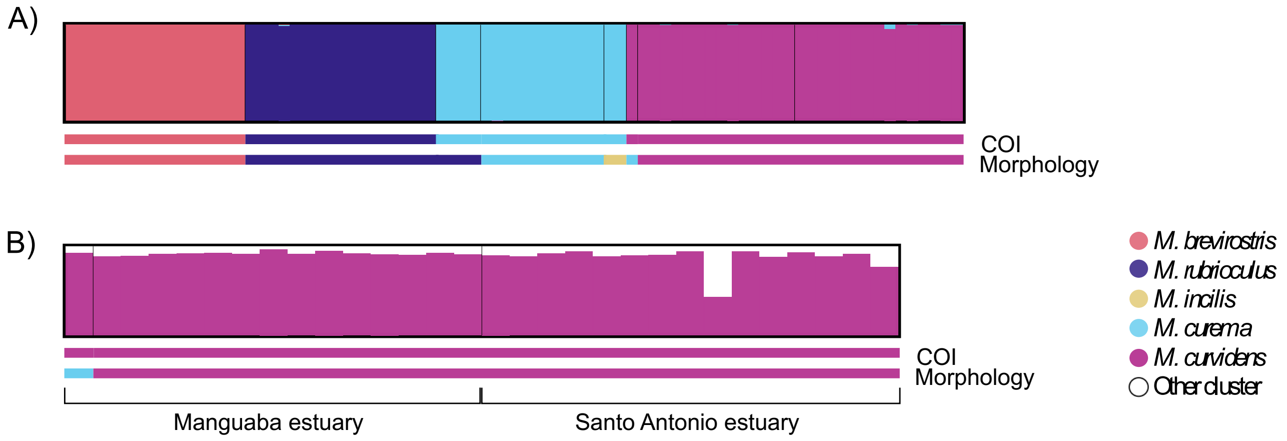

Figure S7. STRUCTURE analyses considering two partitions of the data. A) Analysis performed with 80 *Mugil* individuals (excluding *M. liza* lineage) and a dataset with 0% of missing data (1,879 SNPs). It is evidenced the presence of four homogeneous lineages. B) Analysis performed only with 30 individuals *M. curvidens* individuals sampled at two different estuaries (Manguaba and Santo Antonio) considering 0% of missing data (3,312 SNPs). There is no sign of population genetic structure between the two estuaries at K=2. Bars represent identification of the individuals according to COI gene and morphology.

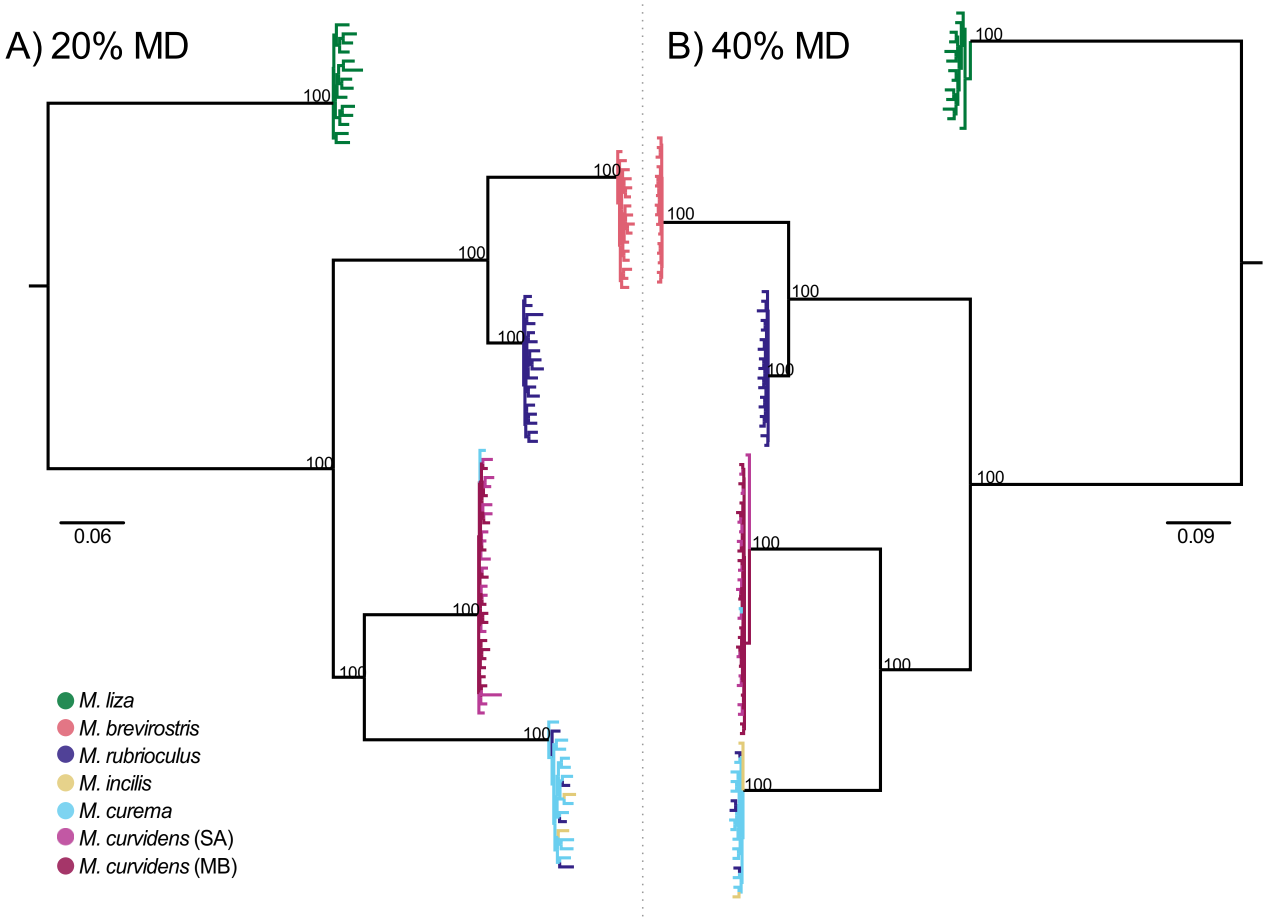

Figure S8. Maximum likelihood phylogenetic tree performed with 1,000 bootstrap replicates and GTRGAMMA model with a dataset considering a maximum of 20% (A) and 40% of missing data (B). Numbers near the nodes represent node support bs. Color of the terminals is according to morphological identification and sample location.

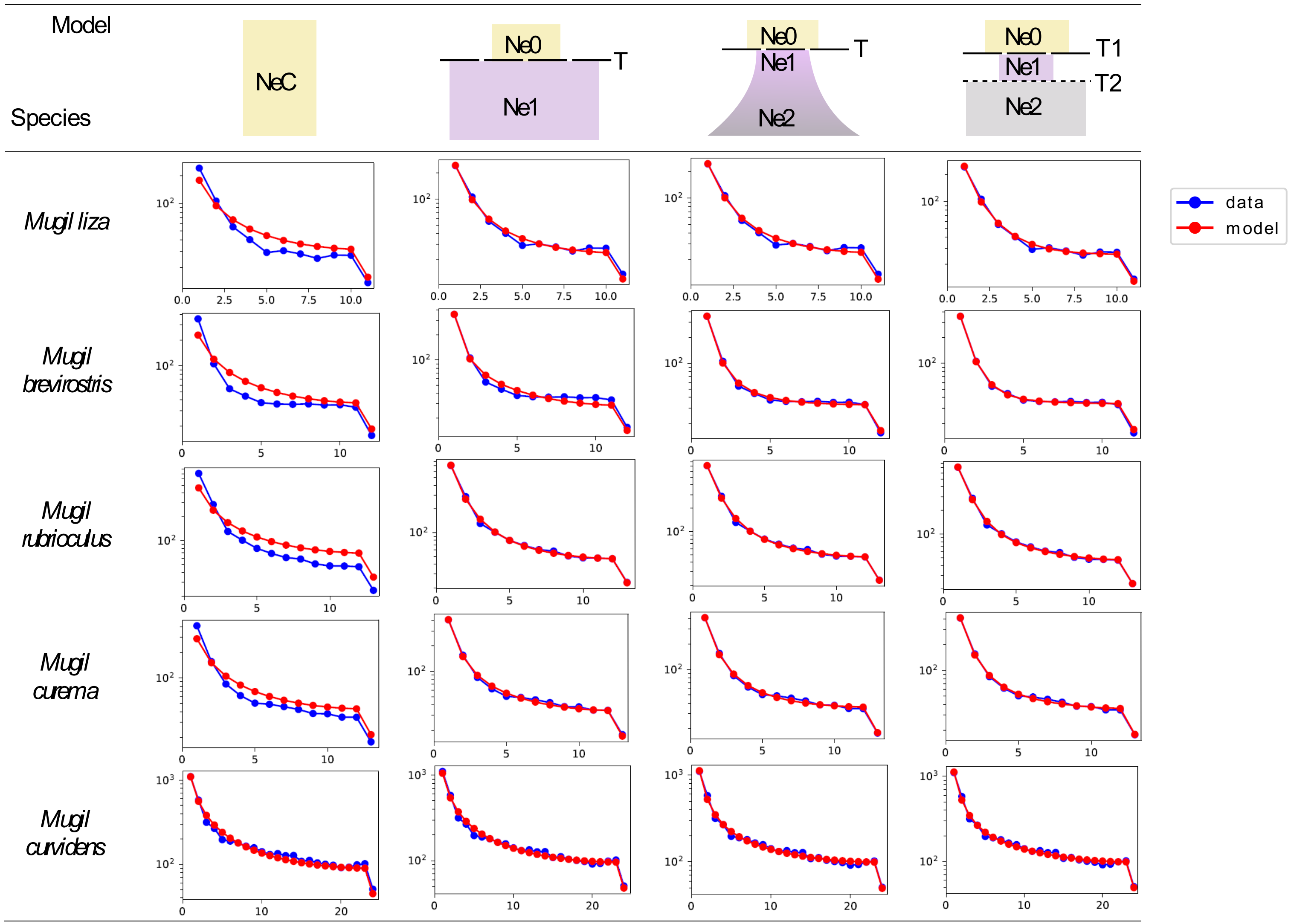

Figure S9. Observed (blue) and simulated (red) site frequency spectra for each *Mugil* species, classified according to genetic clustering. The analyses were based on a dataset allowing 40% of missing data, considering 4 demographic models implemented in δaδi.

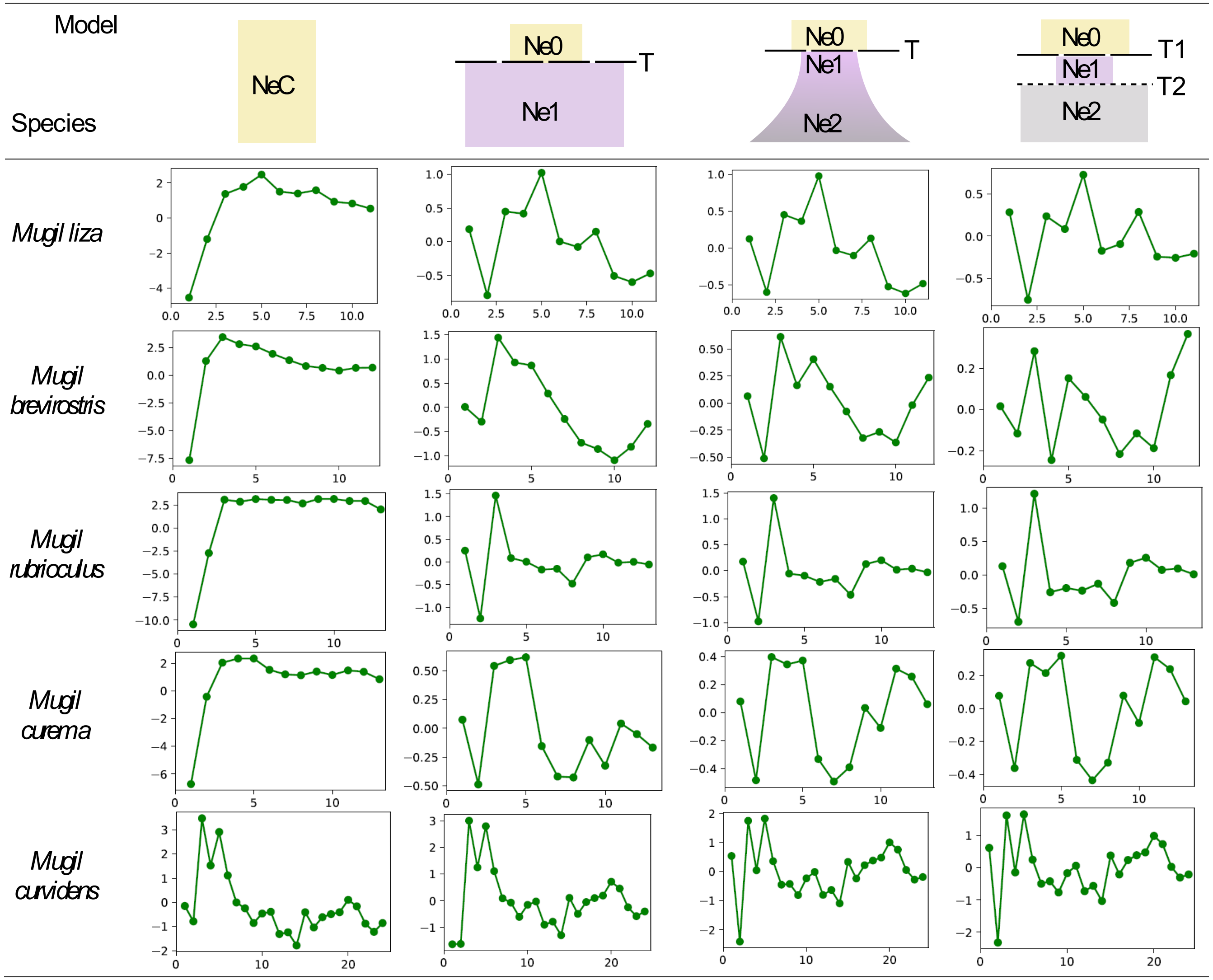

Figure S10. Residuals between observed and simulated site frequency spectra for each *Mugil* species, classified according to genetic clustering. The analyses were based on a dataset allowing 40% of missing data, considering 4 demographic models implemented in δaδi.
